# Chronic oxycodone induces integrated stress response in rat brain

**DOI:** 10.1186/s12868-015-0197-8

**Published:** 2015-09-16

**Authors:** Ruping Fan, Lisa M. Schrott, Stephen Snelling, Julius Ndi, Thomas Arnold, Nadejda L. Korneeva

**Affiliations:** Department of Emergency Medicine, Louisiana State University Health Sciences Center, 1501 Kings Highway, Shreveport, USA; Department of Pharmacology, Toxicology, and Neuroscience, Louisiana State University Health Sciences Center, 1501 Kings Highway, Shreveport, USA; University of Louisiana of Monroe, 700 University Ave., Monroe, LA 71209 USA; Department of Biochemistry and Molecular Biology, Louisiana State University Health Sciences Center, 1501 Kings Highway, Shreveport, USA

**Keywords:** Oxycodone, Morphine, Opioid, Nucleus accumbens, Cortex, Brain stem, Oxidative stress, Nitro-tyrosine, Endoplasmic reticulum stress, Integrated stress response, Hsp70, BiP/GRP78, Phosphorylated eIF2α, Polysomal analysis, Translation, ATF4, PDGFRα, Purkinje cells

## Abstract

**Background:**

Oxycodone is an opioid that is prescribed to treat multiple types of pain, especially when other opioids are ineffective. Unfortunately, similar to other opioids, repetitive oxycodone administration has the potential to lead to development of analgesic tolerance, withdrawal, and addiction. Studies demonstrate that chronic opioid exposure, including oxycodone, alters gene expression profiles and that these changes contribute to opioid-induced analgesic effect, tolerance and dependence. However, very little is known about opioids altering the translational machinery of the central nervous system. Considering that opioids induce clinically significant levels of hypoxia, increase intracellular Ca^2+^ levels, and induce the production of nitric oxide and extracellular glutamate transmission, we hypothesize that opioids also trigger a defensive mechanism called the integrated stress response (ISR). The key event in the ISR activation, regardless of the trigger, is phosphorylation of translation initiation factor 2 alpha (eIF2α), which modulates expression and translational activation of specific mRNAs important for adaptation to stress. To test this hypothesis, we used an animal model in which female rats were orally gavaged with 15 mg/kg of oxycodone every 24 h for 30 days.

**Results:**

We demonstrated increased levels of hsp70 and BiP expression as well as phosphorylation of eIF2α in various rat brain areas after oxycodone administration. Polysomal analysis indicated oxycodone-induced translational stimulation of ATF4 and PDGFRα mRNAs, which have previously been shown to depend on the eIF2α kinase activation. Moreover, using breast adenocarcinoma MCF7 cells, which are known to express the μ-opioid receptor, we observed induction of the ISR pathway after one 24-h treatment with oxycodone.

**Conclusions:**

The combined in vivo and in vitro data suggest that prolonged opioid treatment induces the integrated stress response in the central nervous system; it modulates translational machinery in favor of specific mRNA and this may contribute to the drug-induced changes in neuronal plasticity.

## Background

Oxycodone (6-deoxy-7,8-dehydro-14-hydroxy-3-*O*-methyl-6-oxomorphine) is a strong semi-synthetic opioid. It is prescribed to treat multiple types of pain, especially when other opioids are ineffective. Oxycodone has close structural similarity to morphine and heroin and binds not only μ- but also κ-opioid receptors. Oxycodone’s ability to rapidly cross the blood–brain barrier allows it to reach higher concentrations in the central nervous system (CNS) than in plasma (reviewed in [1]). These properties contribute to oxycodone’s powerful analgesic as well as potential for abuse and addiction. The use of oxycodone in the US increased by 866 % from 1997 to 2007 and accounted for almost half of total opioids dispensed in 2008 [2, 3]. In 2012, the US population consumed over 77 tons of oxycodone [4]. Unfortunately, like other opioids, repetitive oxycodone administration has the potential to lead to development of analgesic tolerance, withdrawal, and addiction. Several studies demonstrate that chronic opioid exposure, including oxycodone, alters gene expression profiles. These changes in gene expression contribute to the opioid-induced analgesic effect, as well as the development of tolerance, and dependence [5–12]. However, little is known about opioids ability to alter the translational machinery in CNS tissues.

Opioids are known to induce clinically significant levels of hypoxia due to respiratory depression, increase intracellular Ca^2+^ levels (reviewed in [13]), and induce the production of nitric oxide (NO) [14], and extracellular glutamate transmission [15]. Moreover, opioid exposure is linked to the induction of oxidative stress in neuronal and non-neuronal tissues, and in cell cultures [16–22] (rev. in [23]). Hypoxia, excessive production of reactive oxygen species (ROS) and NO, increased extracellular glutamate, and induction of endoplasmic reticulum stress by aberrant calcium flow, have all been shown to trigger a defensive mechanism called the integrated stress response (ISR) [24]. The key event in ISR, regardless of the trigger, is phosphorylation of translation initiation factor 2 alpha (eIF2α) that modulates expression and translational activation of specific mRNAs important for adaptation to the stress. In metazoa, there are four kinases that phosphorylate eIF2α: (1) GCN2, general control nonderepressible 2 kinase, which is activated in response to amino acid deprivation, UV irradiation, and proteasome inhibition; (2) HRI, the hemin-regulated inhibitor kinase, that was found to inhibit protein synthesis in heme-deprived lysates and stressed cells; (3) PKR, protein kinase R, which is activated by double-stranded RNA and shuts down translation during viral infection; and (4) PERK, a PKR-like endoplasmic reticulum (ER) kinase, which is activated as part of the unfolded protein response (UPR) to the ER stress [25]. Phosphorylation of eIF2α reduces general translation but allows the ribosome to initiate translation of specific mRNAs containing upstream open-reading frames (uORF) in their 5′UTR via leaky scanning [26], including the transcription factor ATF4 (a cAMP element binding transcription factor) [27, 28]. The expression level of ATF4 and downstream events then determine whether the cell will adapt to the stress condition and continue to grow or will undergo apoptosis. The induction of the ISR in brain tissues due to chronic opioid administration has never been investigated.

We hypothesize that chronic opioid administration triggers the ISR and thus modulates phospho-eIF2α-dependent translation. To test this hypothesis, we used an animal model in which female rats were orally gavaged with 15 mg/kg oxycodone every 24 h for 30 days. In this study we observed that chronic oxycodone exposure induces oxidative/nitrosidative stresses in three areas of the brain: the nucleus accumbens, cortex, and brain stem. Western blotting and immunohistochemical analyses demonstrates that activation of the ISR in oxycodone-exposed brain tissues occurs, inducing over-expression of hsp70 and BiP, increasing phosphorylation of eIF2α and translationally up-regulating the phospho-eIF2α-dependent mRNAs. To investigate whether oxycodone induces ISR in the receptor-dependent manner we treated breast adenocarcinoma MCF7 cells, which are known to express the μ-opioid receptor, with oxycodone for 24 h and demonstrated that activation of the ISR was partially abrogated by pre-treatment of cells with the opioid receptor antagonist naloxone.

## Results

### Daily oxycodone treatment activates signaling pathways characteristic of both acute and chronic opioid response in rat nucleus accumbens

**Fig. 1:**
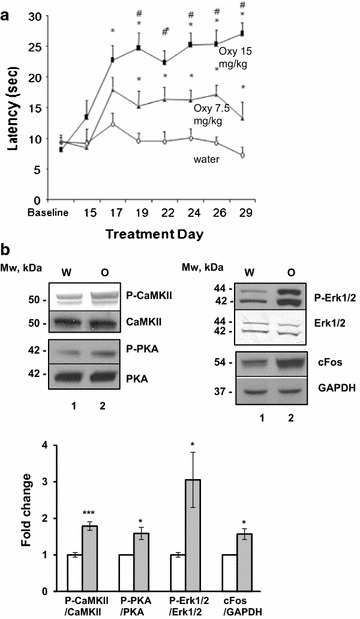
Model for chronic exposure to oxycodone dosing. **a** Hot plate assay to test the antinociceptive effect of oxycodone and the time of tolerance development in rats. Oxycodone (7.5 or 15 mg/kg) or water was administered by oral gavage every 24 h for 29 days. Rats were placed on a hot plate and the time spent until they licked the hind paw was measured. Each point represents a mean value of measurement from 8 animals (±SEM). There was an effect of treatment (F_2, 21_ = 26.08, p < 0.0001), day (F_7, 147_ = 14.03, p < 0.0001) and a treatment × day interaction (F_14, 147_ = 5.38, p < 0.0001). *p < 0.05 vs water; # p < 0.05 vs oxy 7.5 mg/kg (Fisher’s PLSD). *Oxy* oxycodone; *water* vehicle treatment. **b** Western blot analyses of signalling pathways of rewarding circuit in nucleus accumbens. Representative images of western blots of total and phosphorylated CaMKII, total and phosphorylated PKA, total and phosphorylated Erk1/2, cFos, and GAPDH in nucleus accumbens of water (W) and oxycodone (O) treated rats. Below *panel—graphs* representing the densitometric analysis of western blots. The *graphs* represent the mean ratio of phosphorylated protein to corresponding total protein, and mean ratio of cFos to GAPDH. Oxycodone data were normalized to that in water samples from three different drug administration experiments (±SEM). Data was analyzed by Student’s t test. P-CaMKII/CaMKII, p < 0.001; P-PKA/PKA, p < 0.05; P-Erk1/2/Erk1/2, p < 0.05; cFos/GAPGH, p < 0.05. *Open bars* water samples; *gray filled bars* oxycodone samples

First, to determine the antinoceptive effect of oxycodone and development of tolerance in rats, we performed a hot plate assay. Rats were placed on a hot plate and the latency lick the hind paw was measured (Fig. 1a). The latency time for the lower dose, 7.5 mg/kg, of oxycodone-treated rats was nearly double that of untreated rats, while at high dose, 15 mg/kg, the latencies of oxycodone-treated rats increased threefold from 8.1 s to approximately 24 s. The high doze treatment with oxycodone, 15 mg/kg, for 30 days showed development of effective chronic analgesia, similar to that seen in human dosing. Thus, to study the effect of chronic oxycodone exposure, we treated rats with either vehicle (water) or 15 mg/kg oxycodone by oral gavage every 24 h for 30 days.

Because of the potential for the development of abuse and addiction, we investigated the effect of oxycodone on reward signaling in nucleus accumbens of animals sacrificed 2 h after administration of the last dose. It has been found that PKC and CaMKII are responsible for activation of the mitogen activated protein kinase Erk1/2 in nucleus accumbens after acute opioid exposure, while PKA activates Erk1/2 during chronic treatment (reviewed in [13]). Activation of Erk1/2 leads to increased expression of immediate-early genes, including *c*-*fos*, which is known to account for the opioid rewarding effect ([29]; reviewed in [30]). To investigate whether our regiment of treatment induces chronic response, we monitored the level of phosphorylation of CaMKII, PKA, and Erk1/2, as well as the expression level of cFos in nucleus accumbens by western blot analysis. Each sample lysate contained the nucleus accumbens tissue from three rats. The oxycodone data (O) were normalized to the corresponding water-control data (W) from the same treatment experiment. Results are presented on Fig. 1b as the mean of three independent treatment (drug administration) experiments (±SEM). Student’s t test was applied to the data to determine statistical significance. Western blot analysis shows an increase from 1.5- to over 3-fold in the phosphorylation level of CaMKII, PKA, and Erk1/2 kinases in nucleus accumbens lysates of rats treated with oxycodone compared to that of water-treated animals (Fig. 1b). In agreement with Erk1/2 activation, the cFos protein expression increased more than 1.5-fold in the nucleus accumbens of oxycodone-exposed rats (Fig. 1b). These data indicate that the proposed dosing of oxycodone and daily regimen for 30 day produces neuronal activation in the nucleus accumbens that correlates with both acute and chronic signalling leading to rewarding properties.

### Chronic oxycodone treatment induces oxidative and nitrosidative stresses

**Fig. 2:**
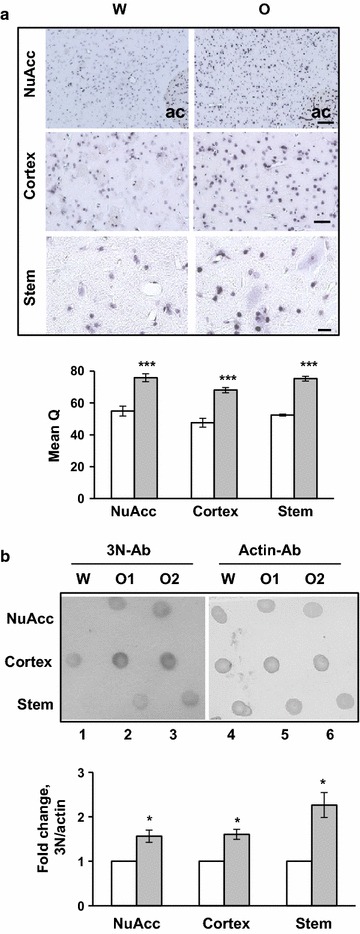
Chronic oxycodone treatment induces biomarkers of oxidative and nitrosidative stresses. **a**. Immunohistochemical analysis of oxidized DNA in nucleus accumbens (NuAcc), cortex, and brain stem of rats treated with water (W) or oxycodone (O). Tissues were stained with antibodies against 8-Hydroxyguanosine (8H). Representative images of area next to anterior commissure (ac) in NuAcc, striatum in cortex area, cerebellum in brain stem area. *Scale bar* denotes: NuAcc, 100 μm; Cortex, 50 μm; Stem, 20 μm. Below the images is statistical analysis of the data. *Open bars* water samples; *gray filled bars* oxycodone samples. The intensity of 8H-Ab stating in nucleus accumbens, cortex, and cerebellum was analyzed using MeanQ method. The *graphs* represent the mean value of signal in three different animals (±SEM). Data was analyzed by Student’s t test. NuAcc, n = 9 fields in water and n = 6 fields in oxycodone samples containing 9–19 cells each, p < 0.001. Cortex, n = 9 fields in water and n = 5 fields in oxycodone samples containing 6–18 cells each, p < 0.001. Stem, n = 5 fields in water and n = 5 fields in oxycodone cerebellums containing 23–51 cells each, p < 0.001. **b** Dot-blot analysis of lysates from nucleus accumbens, cortex, and brain stem of rats treated with water (W) or oxycodone (O1, O2). Two μg of total proteins were spotted on NC membrane and then probed with antibodies against either nitro-tyrosine (3N *left* membrane) or actin (*right* membrane). The 3N signal was normalized to the corresponding actin signal in each sample and then oxycodone data was normalized to the corresponding water data. The* graph* represents the mean ratio of oxycodone to water data obtained in three separate experiments using lysates from different drug administration experiments (±SEM). Data was analyzed by Student’s t test. NuAcc, nucleus accumbens, p < 0.05; Cortex, p < 0.05; Stem, brain stem, p < 0.05. *Open bars* water samples; *gray filled bars* oxycodone samples

To investigate whether chronic oxycodone induces oxidative stress in rat brain, we monitored the level of the 8-Hydroxyguanosine (8H) signal as a marker of oxidative damage in nucleic acids. Immunohistochemical analysis showed a significant increase in staining of 8-H in the nucleus accumbens, cortex, and brain stem areas in oxycodone-exposed rats (Fig. 2a). Increased 8-H staining was observed in the nucleus of various cell types, including neuronal and glial cells, suggesting the induction of oxidative damage to DNA is a systemic effect of chronic oxycodone exposure.

Opioid use is also linked to excessive production of NO [16]. Increased levels of NO and superoxide form peroxynitrite, which modifies tyrosine, produces 3-nitrotyrosine (3N) and leads to protein damage. To investigate whether chronic oxycodone treatment induces protein modification caused by oxidative and nitrosidative stresses we monitored the level of 3-nitrotyrosine in rat brain lysates. Each lysate sample contained corresponding tissues from three rats. The 3N signal was normalized to the corresponding actin signal in each sample and then oxycodone data was normalized to the corresponding water data (Fig. 2b). The graph represents the mean ratio of oxycodone to water data obtained in three separate experiments using lysates from different drug administration experiments (±SEM). Data was analyzed by Student’s t test. Dot-blot analysis shows a significant increase in 3N-Ab immunoreactivity in all three brain lysates: nucleus accumbens, cortex, and brain stem, from oxycodone-exposed animals (Fig. 2b). These data indicate that, like other opioids, prolonged oxycodone treatment induces oxidative and nitrosidative stress in rat brain.

### Oxycodone induces the integrative stress response in rat brain

**Fig. 3:**
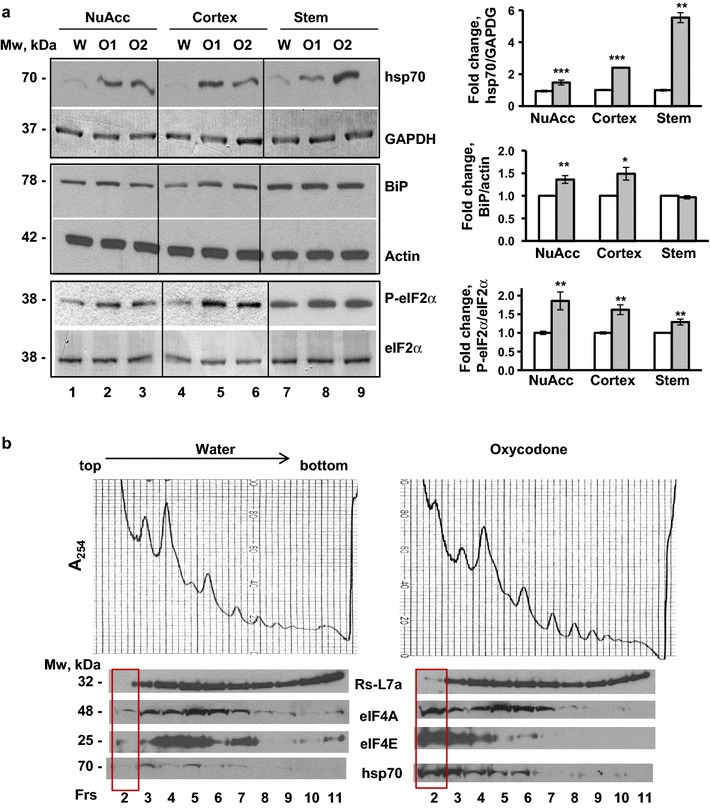
Oxycodone induces integrative stress response in rat brain. **a**. Western blot analysis of hsp70, BiP and phosphorylated eIF2α in rat brain lysates from nucleus accumbens, cortex, and brain stem of rats treated with water (W) or oxycodone (O1, O2). *Left*, the representative images of western blots. *Right*,* graphs* of the densitometric analysis of western blots. The *graphs* represent the mean ratio of signal of hsp70 to GAPDH, BiP to actin, or phospho-eIF2α to total eIF2α with oxycodone data normalized to water samples in corresponding tissues (±SEM). Western blot analysis was performed for each set of proteins using lysates from three separate drug administration experiments and repeated at least twice for each set of lysates. Hsp70, NuAcc, p < 0.001; Cortex, p < 0.001; Stem, p < 0.01. BiP, NuAcc, p < 0.01; Cortex, p < 0.05; Stem, p = 0.38. Phospho-eIF2α, NuAcc, p < 0.01; Cortex, p < 0.01; Stem, p < 0.01. *Open bars* water samples; *gray filled bars* oxycodone samples. **b** Polysomal analyses of nucleus accumbens in sucrose density gradients. *Upper panels* the representative images of polysomal distribution of nucleus accumbens lysates from water and oxycodone-treated animals in sucrose density gradients. Top of the gradient is on *left* side of each image. Direction of sedimentation is shown. The polysomal analysis was repeated three times using lysates from different drug administration experiments. *Lower panels* the representative images of western blots of 60S ribosomal protein L7a (Rs-L7a), eIF4A, eIF4E, and hsp70 in corresponding fractions (Frs) along sucrose gradients. The western blot analysis of proteins was investigated in two sucrose density gradients using lysates from separate drug administration experiments

To investigate whether oxycodone induces the ISR we monitored the expression levels of hsp70 in brain lysates. Hsp70 is one of the major heat shock proteins induced in the central nervous system as part of the general response to a variety of cellular stresses (reviewed in [31]). Real-time PCR analysis of total RNA isolated from nucleus accumbens, cortex and brain stem lysates showed a 7- to 17-fold increase in hsp70 mRNA level in all three tissues from the oxycodone-exposed animals compared to that from the water-treated animals (data not shown). In agreement with real-time PCR data, western blot analysis of lysates from the nucleus accumbens, cortex and brain stem showed an increase in hsp70 protein levels in all three brain areas of oxycodone-treated rats (Fig. 3a, upper panels). Overexpression of hsp70 in the nucleus accumbens, cortex and brain stem lysates suggests that chronic oxycodone administration induces cellular stress in the rat brain. We also observed increased levels of hsp70 in blood samples of oxycodone-exposed animals suggesting a systemic effect of oxycodone (data not shown).

To investigate whether oxycodone induces the unfolded protein response, which is one of the pathways that triggers the ISR, we monitored the expression levels of BiP. Chaperone BiP serves as a sensor of endoplasmic reticulum (ER) stress. Western blot analysis showed significant increase in BiP level in both nucleus accumbens and cortex lysates of oxycodone-treated animals suggesting activation of UPR in these brain areas (Fig. 3a, middle panels). Interestingly, no changes in the BiP levels in brain stem lysates were observed by western blot.

Western blot analysis of brain lysates also demonstrated a significant increase in the phosphorylated translation initiation factor 2α (eIF2α) level in all three tissue lysates from the oxycodone-exposed animals: nucleus accumbens, cortex, and brain stem (Fig. 3a, lower panels). The phosphorylation status of eIF2α serves as a marker of integrated stress response (ISR) that also includes UPR. Since we cannot exclude that, beside endoplasmic reticulum stress, other triggers may contributes to cellular stress response leading to phosphorylation of eIF2α, we suggest that chronic oxycodone treatment induces integrated stress response in rat brain.

Modulation of the translational machinery is a part of the ISR that facilitates cellular adaptation to stress. To investigate the effect that oxycodone has on general translation, we performed ultracentrifugation of lysates from nucleus accumbens in sucrose density gradients. We chose lysate from nucleus accumbens because of the smaller size of the tissue and higher cellular homogeneity compared to other brain tissues containing cortex or brain stem. The polysomal profiles of the nucleus accumbens lysates from water and oxycodone-treated animals did not show significant differences (Fig. 3b, upper panels). Also, the distribution of the large ribosomal subunit (60S) along sucrose density gradients was similar between oxycodone- and water-exposed nucleus accumbens (Fig. 3b, western blot panel, Rs-L7a). However, distribution of the translation initiation factors 4A (eIF4A) and 4E (eIF4E) significantly changed after oxycodone exposure (Fig. 3b, western blot panels, eIF4A and eIF4E). Although, the majority of the translation initiation factors co-sedimented with ribosomal fractions in both water- and oxycodone-exposed tissue lysates (fractions 3 and up), in oxycodone lysate a significant portion of eIF4A and eIF4E also accumulated on the top of the sucrose density gradients (in fraction 2, which contains free proteins complexes and untranslated mRNAs). Interestingly, in oxycodone-treated samples, hsp70 co-sedimented with ribosomal complexes (fractions 3 and up) and also accumulated on the top of the gradient (fraction 2), similar to eIF4A and eIF4E (Fig. 3b, western blot panel, hsp70). Co-sedimentation of hsp70 with ribosomal complexes and translation factors is in agreement with hsp70’s role in co-translational folding of newly synthesized proteins. Sedimentation of hsp70 at the top of the gradient for the oxycodone lysate may indicate accumulation of protein complexes targeted by hsp70 for refolding or proteolysis.

### Oxycodone stimulates translation of the phospho-eIF2α-dependent mRNAs

**Fig. 4:**
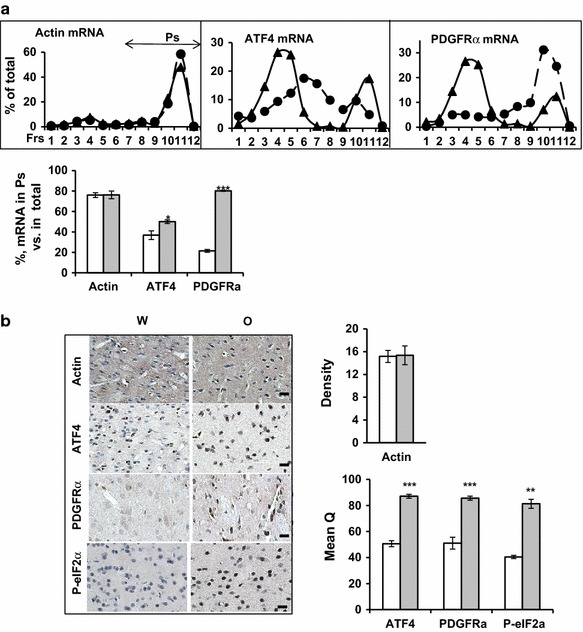
Chronic oxycodone exposure modulates translation in rat nucleus accumbens area. **a** *Upper panels* Real-time-PCR analysis of actin, ATF4, and PDGFRα mRNAs distribution in sucrose density gradients. Nucleus accumbens lysates were ultracentrifuged and fractionated similar to that shown on Fig. 3b. *Filled triangle*, water; *Filled circle*, oxycodone samples. *Graphs* represent percentage of individual mRNA in each fraction. *Lower panel* *graphs* representing percentage of individual mRNA in polysomal complexes (fractions #7–12) relative to the amount of this mRNA in all twelve fractions. The* graphs* represent the mean value from three independent polysomal ultracentrifugations (±SEM). Actin, p = 0.95; ATF4, p < 0.05; PDGFRα, p < 0.001. *Open bars* water samples; *gray filled bars* oxycodone samples. **b** Immunohistochemical analysis of actin, ATF4, PDGFRα, and phosphorylated eIF2α staining in nucleus accumbens areas. *Left* representative images. *Scale bar* denotes 20 μm. *Right* statistical analysis of the data. *Open bars* water samples; *gray filled bars* oxycodone samples. The intensity of actin stating was analyzed using “Density” method, and the intensity of ATF4, PDGFRα, and phospho-eIF2α staining was analyzed using MeanQ method. The *graphs* represent the mean value of intensities measured in brain slides obtained from three animals for each treatment (±SEM). In each slide, at least 3 fields containing 13–74 cells each were investigated. Statistical analysis was performed using Student’s t test. Actin, p = 0.93; ATF4, p < 0.001; PDGFRα, p < 0.001; P-eIF2α, p < 0.01

To investigate the effect that chronic oxycodone treatment has on translation of the mRNAs that are specifically regulated by phosphorylation of eIF2α, we monitored the distribution of ATF4 and PDGFRα mRNAs in the sucrose density gradients similar to that shown on Fig. 3b. The ATF4 mRNA has two upstream open reading frames and is known to be translationally activated by phosphorylation of eIF2α [32]. The PDGFRα mRNA was shown to be translationally de-repressed in a PERK-dependent manner following ER stress in mouse liver [33]. As a control, we monitored polysomal distribution of actin mRNA, which synthesizes a house-keeping protein and is not translationally regulated by activation of the eIF2α kinases [33]. First, we confirmed that oxycodone treatment did not affect the total amount of actin, ATF4, and PDGFRα mRNAs in the nucleus accumbens lysates (data not shown). To investigate the effect that oxycodone has on the translational efficiency of actin, ATF4 and PDGFRα mRNAs we isolated RNA from each fraction of the sucrose density gradients and performed real-time PCR assay using specific primers. In Fig. 4a, the amount of individual mRNA in each fraction is presented as a percentage of the amount of this mRNA in all 12 fractions. To analyze the amount of individual mRNA in the polysomal fractions, representing efficiently translated mRNA (Ps, fractions 7–12), we expressed it as a percentage of mRNA sedimented in all fractions set as 100 % (Fig. 4a, bars below).

We did not observe any changes in actin mRNA distribution along the sucrose density gradient upon oxycodone treatment (Fig. 4a, left panel). Statistical analysis showed that about 80 % of the actin mRNA co-sedimented with polyribosomal complexes (fractions 7–12) in both water and oxycodone nucleus accumbens samples. In contrast, a significant portion of ATF4 mRNA in water-exposed nucleus accumbens lysate sedimented on the top of the sucrose gradients (Fig. 4a, middle panel, fractions 3–5), containing inhibited or slowly-translated mRNAs. However, in oxycodone-exposed nucleus accumbens lysate, ATF4 mRNA accumulated in the heavier part of the gradient, which contains more efficiently translated mRNAs compared to that of the water-treated samples (fractions 6–12). Interestingly, oxycodone exposure shifted a fraction of the ATF4 mRNA from the heavy complexes (water sample, fractions 9–12) to the middle part of the gradient, suggesting changes in the rate of translation for this mRNA. Statistical analysis showed an increase in the amount of ATF4 mRNA that co-sedimented with the polyribosomal complexes (fractions 7–12) in oxycodone nucleus accumbens lysates (50 ± 1.8 %) compared to that of the water-treated lysates (37 ± 4.3 %). The distribution of PDGFRα mRNA along sucrose density gradients also changed dramatically after oxycodone exposure. Real-time PCR analyses showed that in the nucleus accumbens of the water-treated animals, the majority of mRNA accumulated in fractions 2–6 containing inhibited or slowly-translated mRNAs (Fig. 4a, right panel). However, in the oxycodone-exposed nucleus accumbens, the majority of PDGFRα mRNA accumulated in the heavy polyribosomal complexes (fractions 9–12), containing efficiently translated mRNAs. Statistical analysis showed an increase in the amount of PDGFRα mRNA that co-sedimented with the polyribosomal complexes (fractions 7–12) in oxycodone-treated nucleus accumbens lysate (80 ± 0.5 %) compared to that of water-treated lysate (21 ± 1.3 %). These results suggest that chronic oxycodone exposure stimulated translation of ATF4 and PDGFRα mRNAs in the rat brain areas containing the nucleus accumbens.

To confirm translational activation of ATF4 and PDGFRα mRNAs by chronic oxycodone treatment we monitored the ATF4 and PDGFRα protein expression in nucleus accumbens and compared it to the actin level. Immunohistochemical staining showed no change in actin protein levels after oxycodone treatment (Fig. 4b). In contrast, the ATF4 and PDGFRα protein levels increased almost twofold in the nucleus accumbens area in animals treated with oxycodone. Interestingly, this brain area also demonstrated increased staining for phospho-eIF2α (Fig. 4b) and oxidized DNA (Fig. 2a), confirming the induction of integrated stress response in oxycodone-exposed nucleus accumbens.

### Oxycodone differentially activates the ISR in various brain areas

**Fig. 5:**
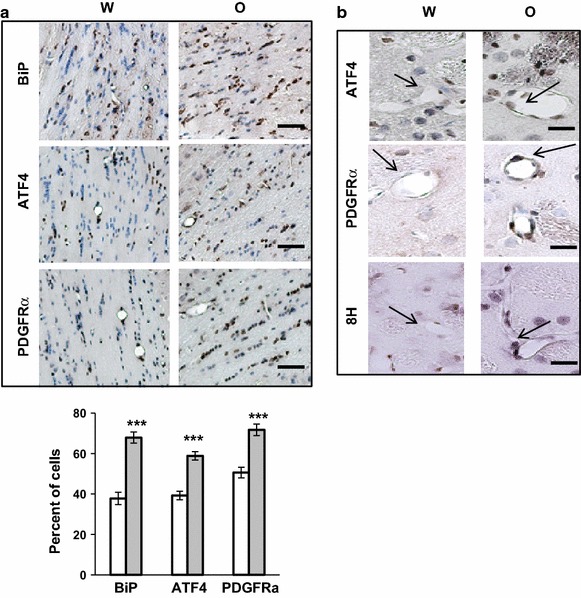
Oxycodone increases ATF4 and PDGFRα expression in corpus collosum and striatum. **a** Immunohistochemical analysis of BiP, ATF4, and PDGFRα in corpus collosum. *Upper panels* representative images of corpus collosum areas of rats treated with water (W) or oxycodone (O). *Scale bar* denotes 50 μm. *Lower bars* statistical analysis of the data. *Open bars* water samples; *gray filled bars* oxycodone samples. The *graphs* represent the mean value of percentage of cells expressing BiP, ATF4, or PDGFRα in corpus collosum areas obtained from three animals (±SEM). In each slide, at least 5 fields containing 25–70 cells were investigated. Statistical analysis was performed using Student’s t test. BiP, p < 0.001; ATF4, p < 0.001, PDGFRα, p < 0.001. **b** Immunohistochemical analysis of ATF4, PDGFRα, and oxidized DNA (8H) in striatum areas of rats treated with water (W) or oxycodone (O). *Scale bar* denotes 20 μm. *Arrows* point to the blood vessels

To investigate whether chronic oxycodone induces the ISR biomarkers in other brain areas we monitored the BiP, ATF4, and PDGFRα expression in corpus collosum, striatum, cerebral cortex, hippocampus, and cerebellum. In corpus collosum, immunohistochemical analysis revealed that chronic oxycodone administration increased percentage of cells expressing BiP, ATF4, and PDGFRα by 1.7-, 1.5-, and 1.4-fold respectively (Fig. 5a). This area also demonstrated induction of the oxidative stress as monitored by 8-Hydroxyguanosine signal (data not shown). Interestingly, in water animals, 30–50 % of cells in corpus collosum showed expression of BiP, ATF4, and PDGFRα (Fig. 5a, white bars) suggesting activation of the integrated stress response in cells in white matter area even under normal conditions. This could be a result of endoplasmic reticulum stress due to neuronal activity. Induction of oxidative stress by chronic oxycodone treatment may further increase stress level in these cells and, thus, stimulate expression of the stress-response proteins such as BiP and ATF4. In striatum, the level of ATF4 and PDGFRα expression also increased after chronic oxycodone exposure (Fig. 5b). Cells lining the blood vessels (arrow) showed increased staining for both proteins, ATF4 and PDGFRα, as well as oxidized DNA (8H), suggesting induction of the ISR in these cells in response to oxidative stress following oxycodone treatment. This result also indicates that the ISR may affect the blood–brain barrier cellular composition during oxycodone administration.

**Fig. 6:**
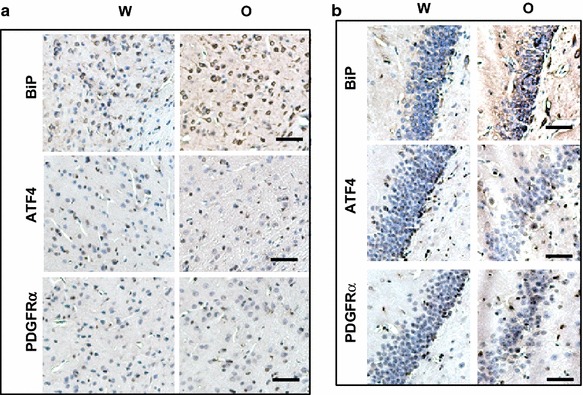
Oxycodone increases BiP but not ATF4 and PDGFRα expression in cerebral cortex and hippocampus. **a** Immunohistochemical analysis of BiP, ATF4, and PDGFRα in cerebral cortex of rats treated with water (W) or oxycodone (O). *Scale bar* denotes 50 μm. **b** Immunohistochemical analysis of BiP, ATF4, and PDGFRα in hippocampus of rats treated with water (W) or oxycodone (O). *Scale bar* denotes 50 μm

In cortical areas, immunohistochemical analysis showed almost twofold increase in expression of BiP, mostly in pyramidal cells, but no difference in ATF4 and PDGFRα staining after oxycodone treatment (Fig. 6a). Similarly, oxycodone increased the percentage of cells expressing BiP in hippocampal areas, but did not affect ATF4 and PDGFRα expression levels (Fig. 6b). These data suggest that in specific brain areas, such as cerebral cortex and hippocampus, prolong oxycodone administration induces endoplasmic reticulum stress without activation of the integrated stress response.

**Fig. 7:**
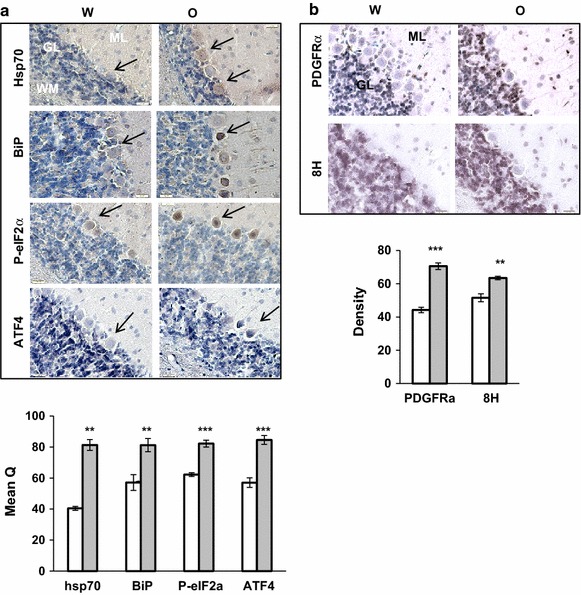
Oxycodone increases ATF4 in purkinjie cells and PDGFRα expression in granular layer of cerebellum. **a** Immunohistochemical analysis of hsp70, BiP, phosphorylated eIF2α, and ATF4 in Purkinje cells. *Upper panels* representative images of Purkinje cells (*arrow*) of rats treated with water (W) or oxycodone (O). *Scale bar* denotes 20 μm. *WM* white matter; *GL* granule layer; *ML* molecular layer. *Lower bars* statistical analysis of the data. *Open bars* water samples; *gray filled bars* oxycodone samples. The intensity hsp70, BiP, phosphorylated eIF2α, and ATF4 staining in Purkinje cells was analyzed using the Q method. The *graphs* represent the mean value of intensities measured in brain slides obtained from three animals for each treatment (±SEM). In each slide, at least 3 fields containing 6–18 Purkinjie cells each were investigated. Statistical analysis was performed using Student’s t test. Hsp70, p < 0.01; BiP, p < 0.01; P-eIF2α, p < 0.001; ATF4, p < 0.001. **b** *Upper panels* immunohistochemical analysis of PDGFRα and oxidized DNA (8H) in granular layers (GL) of rats treated with water (W) or oxycodone (O). *Scale bar* denotes 20 μm. *ML* molecular layer. *Lower bars* statistical analysis of the data. *Open bars* water samples; *gray filled bars* oxycodone samples. The intensity of PDGFRα and oxidized DNA staining in granule cells was analyzed using Density method. The *graphs* represent the mean value of intensities measured in brain slides obtained from three animals for each treatment (±SEM). In each slide, at least 3 fields were investigated. Data was analyzed by Student’s t test. PDGFRα, p < 0.001; 8H, p < 0.01

In the cerebellum of oxycodone treated animals, the ISR biomarkers: hsp70, BiP, phosphorylated eIF2α, and ATF4, were all localized in Purkinje cells, suggesting activation of endoplasmic reticulum stress and induction of the ISR by chronic oxycodone (Fig. 7a). In contrast, the majority of the PDGFRα staining increased in granular and molecular layers of oxycodone-exposed tissues, similar to that of oxidized DNA staining (Fig. 7b). This result suggests that PDGFRα expression may also be triggered by a mechanism other than the ISR during oxycodone exposure.

### Oxycodone activates the ISR in MCF-7 cells

To investigate whether oxycodone induces the integrative stress response in an opioid receptor-dependent manner, we used breast adenocarcinoma MCF7 cells, which are known to express the μ-opioid receptor. First, we pre-treated cells with either water or 10 μM the opioid receptor antagonist naloxone for 15 min and then added 5 μM oxycodone or vehicle and incubated the cells for another 24 h. We did not observe morphological changes in cells treated with oxycodone alone or in combination with naloxone (data not shown).

**Fig. 8:**
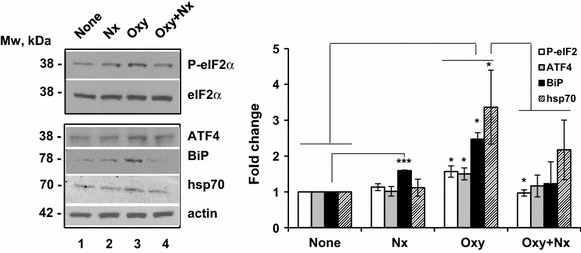
Oxycodone activates the integrated stress response in MCF7 cells. Western blot analysis of phosphorylated and total eIF2α, ATF4, BiP, hsp70, and actin in MCF7 cell lysates treated with water and vehicle (None), 10 μM naloxone and vehicle (Nx), water and 5 μM oxycodone (Oxy) or combination of 10 μM naloxone and 5 μM oxycodone (Oxy + Nx). *Left* the representative images of western blots of corresponding proteins. *Right* *graphs* of the densitometric analysis of western blots. The *graphs* represent the mean ratio of signal of phosphorylated to total eIF2α, and ATF4, BiP, and hsp70 to actin obtained from three individual experiments using separate cell cultures. “Oxy”, “Nx”, and “Oxy + Nx” data were normalized to the “None” value set as one (±SEM). To determine statistical significance, data was analyzed by Student’s t test: Oxy vs None: P-eIF2α, BiP, ATF4, and hsp70, p < 0.05. Nx vs None: BiP, p < 0.001. Oxy ± Nx vs Oxy: P-eIF2α, p < 0.05; BiP, ATF4, and hsp70, p > 0.18

Western blot analyses revealed a significant increase in phopsho-eIF2α, ATF4, BiP, and hsp70 staining after incubation with oxycodone, suggesting the induction of endoplasmic reticulum stress and activation of the ISR (Fig. 8, lane 3 vs 1, “Oxy” vs “None”). Pre-treatment of cells with naloxone completely abrogated the increase in phosphorylation of eIF2α by oxycodone, suggesting that prolonged oxycodone exposure induces the ISR in the receptor-dependent manner (eIF2α, lane 4 vs 3, “Oxy + Nx” vs “Oxy”). The ATF4, BiP, and hsp70 levels in cells pre-treated with naloxone followed by incubation with oxycodone varied significantly from experiment to experiment, suggesting that other factors may contribute to the stress response in a receptor-independent manner (lane 4 vs 3, “Oxy + Nx” vs “Oxy”). Interestingly, BiP protein levels also significantly increased in cells incubated with naloxone alone suggesting that either treatment, prolong agonist (oxycodone) or antagonist (naloxone), induces endoplasmic reticulum stress in these cells (BiP, lanes 2 or 3 vs 1, “Nx” or “Oxy” vs “None”). However, naloxone-induced endoplasmic reticulum stress was not sufficient to activate the ISR since naloxone alone failed to increase phosphorylation of eIF2α or overexpression of ATF4 and hsp70 (lane 2 vs 1, “Nx” vs “None”). These data suggest that prolonged oxycodone treatment induces endoplasmic reticulum stress and the ISR.

## Discussion

To investigate the effect of chronic oxycodone administration, we treated female 60 day-old Sprague–Dawley rats with 15 mg/kg oxycodone or water by oral gavage every 24 h for 30 days. We have previously demonstrated that this model is well-tolerated by the rats [34, 35]. Some tolerance does develop, but there are no measurable physiological indices of dependence and withdrawal with the continual dosing regimen. The dosing regimen provides an opioid level leading to antinociception, which makes it a clinically relevant model. If there is stress associated with the model, we feel that it would be similar to that in a human situation.

In this study, we demonstrated that the integrated stress response (ISR) is induced by chronic oxycodone administration in rats and cell culture models. The ISR is activated by various triggers, allowing cells to adapt to changing environments or to initiate programmed cell death. Hypoxia, endoplasmic reticulum stress due to aberrant Ca^2+^ flow, or the induction of excitotoxic stress, are all associated with opioid abuse. The ISR is manifested by overexpression of several factors including hsp70. Indeed, previously it was found that morphine induces expression of hsp70 in the rat amygdala [36], frontal cortex [5, 37], locus coeruleus [8], as well as in cultured hippocampal neurons [38]. Moreover, it was demonstrated that morphine increases the hsp70 mRNA level in rat brain in the temperature-independent manner [37] supporting the idea of stress as a major trigger of the opioid-induced hsp70 expression. In this study, we also demonstrate that chronic oxycodone administration leads to an increase in both hsp70 mRNA and protein levels in brain lysates from the nucleus accumbens, cortex, and brain stem. Moreover, ultracentrifugation of the nucleus accumbens lysates from oxycodone-treated animals in sucrose density gradients shows co-sedimentation of hsp70 with translational complexes that included ribosomes, translation initiation factors eIF4E and eIF4A, and mRNAs, in agreement with the hsp70 role in co-translational protein folding. We also observed sedimentation of hsp70 on the top of the gradient possibly with unfolded proteins.

Our observation that chronic oxycodone administration induced expression of hsp70 in various rat brain areas is in contrast to several reports that did not find evidence for the induction of hsp70 expression after opioid treatment. One study investigating the effect of chronic oxycodone treatment on the gene expression profile in whole rat brain did not detect changes in the expression level of hsp70 [9]. This discrepancy could be due to the fact that very often the microarray protocol involves enrichment of mRNA fraction via purification with oligo(dT) magnetic beads. However, since hsp70 mRNA lacks the poly(A)-tail, it will be lost during sample preparation. In another study using human embryonic kidney 293 cells expressing MOR1, a 2-h treatment with morphine did not increase Hsp70 mRNA expression [37]. In the study presented here, we demonstrate induction of hsp70 expression in MCF7 cells after 24-h treatment with oxycodone, suggesting that late events such as the accumulation of cellular damage trigger hsp70 overexpression by opioid exposure. Moreover, pre-treatment of MCF7 cells with naloxone did not completely abolish the increase in hsp70 levels by oxycodone supporting the idea that late events may trigger cellular stress in a receptor-independent manner. One such event could be endoplasmic reticulum (ER) stress. Our and others data suggest that excessive or chronic opioid administration induces ER stress leading to activation of the unfolded protein response (UPR). One study demonstrated that chronic morphine treatment induced neuronal pathological changes in rough endoplasmic reticulum in the ventral tegmental area [39]. In another study, morphine administration caused about twofold increase in BiP gene level, a biomarker of UPR, in rat frontal cortex [5]. We observed BiP protein overexpression in various brain areas after chronic oxycodone exposure, including the nucleus accumbens, corpus collosum, cerebral cortex, and brain stem. We also demonstrated induction of the BiP in MCF7 cells after 24-h oxycodone treatment. Naloxone alone also led to increased BiP protein expression suggesting that both antagonist and prolonged agonist treatments are capable to induce endoplasmic reticulum stress.

In neurons, activation of BiP by calcium mobilization or accumulation of unfolded/damaged proteins in ER may lead to phosphorylation of the PKR-like endoplasmic reticulum kinase (PERK) and its downstream target, translation initiation factor eIF2α, and these initiate the UPR. In addition, phosphorylation of eIF2α is also triggered by a variety of other stresses including hypoxia, excessive production of ROS and NO or increase in extracellular glutamate that activates defensive mechanism called integrated stress response (ISR) [24]. In general, phosphorylation of eIF2α leads to the attenuation of global protein synthesis but stimulation of translation of specific mRNAs containing upstream open-reading frames (uORF) in their 5′UTR via leaky scanning [26]. Such an approach allows cells to reduce the load of misfolded proteins in ER but stimulates expression of proteins necessary for survival during stress. The ATF4 mRNA has two uORFs and was shown to be translationally up-regulated by phosphorylation of eIF2α [32]. The ATF4 is transcription factor that regulates down-stream genes expression in response to variety of stresses.

The effect of opioid exposure on activation of integrated stress response including phosphorylation of eIF2α and overexpression of ATF4 has never been investigated. We demonstrated increased phosphorylation of eIF2α and translational up-regulation of the ATF4 mRNA in various areas of rat brain after chronic oxycodone treatment including the nucleus accumbens, corpus collosum, striatum, and brain stem. Interestingly, in Purkinje cells, hsp70, BiP, phosphor-eIF2α, and ATF4, were all localized in cell bodies suggesting induction of ER stress and activation of the ISR by chronic oxycodone exposure. We also demonstrated overexpression of hsp70, BiP, and ATF4 and also increased phosphorylation of eIF2α in MCF7 cells after 24-h incubation with oxycodone suggesting the direct link between opioid exposure and induction of the ISR. Interestingly, phosphorylation of eIF2α was abrogated by pretreatment of cells with naloxone suggesting the μ-opioid receptor-dependent mechanism of the oxycodone-mediated eIF2α phosphorylation. However, the rest of ISR members: hsp70, BiP, and ATF4 demonstrated fluctuation in response to oxycodone after pre-treatment of cells with naloxone, suggesting that part of the stress response in these cells is receptor- and phospho-eIF2α-independent.

We also demonstrated translational upregulation of PDGFRα mRNA and increased PDGFRα protein expression in several oxycodone-exposed brain areas. Previously, PDGFRα mRNA was identified as one of the mRNAs that shifted into polysomes in the eIF2α kinase, PERK, activation-dependent manner [33]. We observed localization of PDGFRα and the ISR machinery, BiP, phopsho-eIF2α, and ATF4 in nucleus accumbens, striatum, and cerebellum. Some cells adjacent to the blood vessels in oxycodone-exposed brains demonstrated increased staining for ATF4, PDGFRα, and oxidized DNA, suggesting induction of the ISR in cells controlling the blood–brain barrier (BBB) integrity. Previously it was shown that activation of PDGFRα may contribute to altered BBB permeability as a compensatory effect to increase oxygen flow [40, 41]. Indeed, a connection between opioid exposure and BBB impairment has been described in several studies. For example, human brain microvascular endothelial cells (HBMEC) treated with morphine induces production of PDGF that lead to disruption of BBB [42]. In a rat model, withdrawal after chronic morphine administration resulted in increased BBB permeability [43] that was markedly attenuated by pretreatment with an antioxidant H-290/51 [44], suggesting a link to the oxidative stress.

Thus, we observed increase in expression of biomarkers of the endoplasmic reticulum stress (BiP) and integrated stress response (ATF4) in nucleus accumbens, corpus collosum, striatum, and Purkinjie cells in rats treated with 15 mg/kg oxycodone for 30 days. However, in cortical and hippocampal areas prolong oxycodone treatment stimulated expression of BiP without activation of the ISR suggesting different than endoplasmic reticulum rout of stress induction by oxycodone. Since cells in these brain areas expressed BiP at lower level even in the water-treated animals, it is possible that this pro-survival pathway promotes better adaptation of the neuronal cells to more severe stress.

The link between other drugs of abuse and induction of the ISR in brain has been reported. Large doze of methamphetamine induced endoplasmic reticulum stress and expression of hsp70, BiP, ATF4, and other stress-response biomarkers in rat striatum after just 1 day of treatment [45]. Interestingly, co-administration of methamphetamine with a putative dopamine receptor D1-agonist, SCH23390, completely blocked expression of these proteins suggesting a receptor-dependent mechanism of the ISR induction. Increase in ATF4 expression was demonstrated in response to amphetamine administration in rats [46]. The ATF4 transcriptional activity was shown to contribute to the post-synaptic terminal and dendritic spine morphology [47] suggesting the mechanism of the opioid-induced modulation of neuronal plasticity. Methamphetamine [43, 48] and cocaine, have been also shown to induce BBB leakage [49]. Thus, our and other’s studies suggest that drugs of abuse may activate the integrated stress response in specific brain areas. The members of the ISR machinery may serve as targets for the therapeutic protection against drug-induced neuronal impairment and the BBB disruption.

Active protein synthesis is important for neuronal plasticity [50]. Further study needed to investigate how drug abuse modulates translational machinery and whether these changes contribute to development of tolerance, addiction, or pain perception. Overall, this study suggests that prolonged opioid treatment affects translational machinery via modulation of activity of the translation factors that affects translation of specific mRNA and thus may contribute to the changes in neuronal plasticity during drug abuse.

## Conclusions

Our study found that chronic oxycodone administration triggers induction of oxidative and nitrosidative stresses in rat brain. We demonstrated activation of the integrated stress response: overexpression of hsp70, BiP, and ATF4 and also increased phosphorylation of eIF2α, in rat brain and MCF7 cell culture by prolonged oxycodone treatment. Moreover, we found that prolonged opioid treatment affects translational machinery suggesting the mechanism of opioid-induced changes in neuronal plasticity during drug abuse that contribute to development of tolerance, addiction, or pain perception. Our report documenting increased expression of PDGFRα in brain tissues after chronic oxycodone treatment also suggests a new target for the therapeutic protection against drug-induced BBB disruption.

## Methods

### Animal model

Female 60 day-old Sprague–Dawley rats (180–240 g) were purchased from Harlan Indianapolis, IN. They were fed chow and water ad libitum and maintained on a 12-h light/dark cycle. The animals were housed three to a cage and allowed to acclimate for at least 1 week before experiments were conducted. The protocol for animal studies was approved by the Louisiana State University Health Science Center, Institutional Animal Care and Use Committee. Rats were assigned to one of two groups (*n* = *3*/group) administered either oxycodone (Mallinckrodt Inc., St. Louis, MO, USA) or its vehicle water. Oxycodone (15 mg/kg) Fisher’s PLSD or water was administered by oral gavage (volume of 1.0 ml/kg) every 24 h for 30 days. The oxycodone-treated group was compared directly to the water-treated control group, which was handled, treated, and sacrificed at the same time and under the same conditions. For statistical analysis this treatment [three water- (W) and six oxycodone-treated (O1 and O2) animals] was repeated three times. To determine the toxicity of the oxycodone, rats were weighed daily. There was no significant weight loss in either group of animals. In addition, anti-nociceptive effect of 15 mg/kg of oxycodone treatment was assessed by recording the latency to lick the hindpaws when the rat was placed on a hotplate (52 °C; maximal latency 30 s) over 30 days of treatment.

### Lysates preparation

For Western blot and dot-blot analyses, rats in both groups were sacrificed by CO_2_ asphyxiation and decapitated 2 h after administration of the last dose of oxycodone or the water vehicle. Whole-brain tissues were harvested, and then tissues containing cortex, nucleus accumbens and brain stem were collected in separate tubes containing ice-cold buffer A (50 mM Tris–HCl, 140 mM NaCl, 5 mM KCl, 6 mM MgCl_2_, and 1 mM Na-vanadate pH 8.0). The corresponding tissues from three rats were pooled together in order to obtain enough material for all analyses. Thus, each sample contained tissues from three animals. Tissues were homogenized in ice-cold buffer B [100 mM Tris–HCl, 150 mM KCl, 10 mM MgCl_2_, 200 mM Sucrose, 5 mM DTT, 0.5 mg/ml heparin, 0.5 % Triton X-100, 0.5 % NP-40, and 0.5 % deoxyholate, Complete™ EDTA-free protease inhibitor cocktail (Roche), and phosphatase inhibitor cocktails 2 and 3 (Sigma)], by 30 strokes with a Teflon/glass homogenizer, incubated on ice for 10 min, pipetted 10 times with 1 ml tip and centrifuged at 10,000×*g* at 4 °C for 5 min. The supernatants were collected and concentration of proteins in the supernatant was measured with Micro BCA Protein Assay Kit (Thermo Scientific Pierce). Brain tissue lysates (cortex, nucleus accumbens, and brain stem) were stored at −80 °C until further processing. We choose to investigate these three brain areas based on their functions that may contribute to pain perception and also cognitive and motor impairment (cortex); to reward and the abuse potential (nucleus accumbens); and to mediation of respiratory depression and some of the ant-nociceptive and pro-emetic effects (brain stem).

### Western blot analysis of brain lysates

To analyse the expression of proteins in brain tissues, equal amounts of total protein were loaded on a 12 % or 4–12 % NuPAGE^®^ Novex^®^ Bis–Tris Gel (Invitrogen). The Full-Range Rainbow protein molecular weight marker (GE Healthcare Life Science) was loaded on the same gel to identify the position of specific proteins. Proteins were separated by SDS-PAGE gel and then transferred to a Nitrocellulose membrane (Bio-Rad) using a Mini Trans-Blot cell (Bio-Rad). Expression of specific proteins were determined by probing the membrane with antibodies against P-CaMKII (Thr286) (dilution 1:1000), CaMKII (pan) (dilution 1:1000), P-Erk1/2 (Thr202/Tyr204) (dilution 1:4000), Erk1/2 (137F5) (dilution 1:8000), P-PKA Cα (Thr197) (dilution 1:2000), and PKA Cα (4782) (dilution 1:8000), P-eIF2α (Ser51) (dilution 1:2000), eIF2α (D7D3) XP (dilution 1:4000), HSP70 (D69) (dilution 1:1000) all from Cell Signalling; cFos (dilution 1:4000) from Santa Cruz; GRP78 (BiP) (dilution 1:8000) from Abcam; actin (dilution 1:8000) from Sigma; and GAPDH (dilution 1:8000) from Fitzgerald. Mouse monoclonal anti-eIF4A antibody (dilution 1:2000) was a gift from Dr. Hans Trachsel, Bern, Switzerland. Rabbit anti-eIF4E antibody (dilution 1:1000) was a gift from Dr. Robert E. Rhoads, LSUHSC. The membranes were incubated with primary antibodies in 5 % BSA in buffer TBS-T (20 mM Tris–HCl, 150 mM NaCl, and 0.1 % Tween 20, pH 7.5) overnight at 4 °C, washed three times for 15 min with TBS-T, and incubated for 1 h at room temperature with anti-mouse secondary antibodies (for GAPDH and eIF4A antibodies) or anti-rabbit secondary antibodies (for all other primary antibodies) conjugated with horse-peroxidase (Vector Laboratories, Inc.) in 5 % non-fat dry milk in TBS-T. Blots were developed with the Western Lightning ECL Pro development kit (PerkinElmer) and exposed to HyBlot CL autoradiography film (Denville Scientific). Quantitative analysis of Western blot images was performed using the ImageQuant TL software (GE Healthcare Life Science). Each sample contained the corresponding brain tissue from three rats. The oxycodone data (O1 or O2) were normalized to the corresponding water-control data (W) from the same treatment experiment. Results are presented as the mean of three independent treatment (drug administration) experiments ± SEM. Student’s t test was applied to the data to determine statistical significance, and data with p value lower than 0.05 was considered to be statistically different.

### Dot-blot and statistics analysis

Two micrograms of total lysates from nucleus accumbens, cortex, and brain stem of animals treated with water or oxycodone were spotted on Nitrocellulose membrane. After samples dried, membranes were blocked and probed with primary and secondary antibodies as described for western blot analysis. The level of nitro-tyrosine in samples was determined using mouse antibodies against 3-nitrotyrosine (39B6) (diluted 1:5000) from ENZO and anti-mouse secondary antibodies conjugated with horse-peroxidase (Vector Laboratories, Inc.) in 5 % non-fat dry milk in TBS-T. 3-nitrotyrosine signals were detected using the Western Lightning ECL Pro development kit (PerkinElmer). The same membranes were stripped, blocked and then incubated with rabbit anti-actin antibodies (dilution 1:5000) from Sigma and then with anti-rabbit alkaline-phosphatase conjugated secondary antibodies (Vector Laboratories, Inc.). Actin signal was visualized using BCIP/NBT color development substrate (Promega, Madison, WI, USA). Quantitative analysis of dot-blot images was performed using the ImageQuant TL software (GE Healthcare Life Science). Each membrane contained one water and two oxycodone samples from the same drug administration experiment. This experiment was repeated three times using lysates from different drug administration experiments. The 3N signal was normalized to the corresponding actin signal in each sample and then oxycodone data was normalized to the corresponding water data, such as (3N_oxy_/Actin_oxy_)/(3N_water_/Actin_water_). The graph represents the mean ratio of oxycodone to water data (±SEM). To determine statistical significance, data was analyzed by Student’s t test, and data with p value lower than 0.05 was considered to be statistically different.

### Immunohistochemical analysis

For immunohistochemical analyses, three rats treated with water and three rats treated with oxycodone were anesthetized with injection of 65 mg/kg i.p. of sodium pentobarbital and then perfused through the aortic arch with 100 ml ice-cold saline followed by 400 ml ice cold 4 % paraformaldehyde in 0.1 M sodium phosphate buffer, pH 7.4 (PB). The whole brain was removed and placed in 4 % paraformaldehyde in PB overnight at 4 °C, transferred to a 15 % sucrose in 0.1 M PB for 24 h, then to 30 % sucrose in PB for 24 h and then stored in 70 % ethanol at 4 °C until further processing. Rat brain sections containing cortex and nucleus accumbens (plates 12-30, Rat Brain Atlas, Paxinos and Watson) or brain stem (plates 122 and later, Rat Brain Atlas, Paxinos and Watson) were embedded in paraffin according to a standard protocol, cut into 10 μm thick slices and then mounted on glass slides, two or three consecutive slices on one slide (Millennia 1000). Tissue sections were deparaffinized by warming in the oven at 65 °C for 1 h, rehydrated in xylene, 100 % and 95 % solutions of ethanol and then in water. The antigens were retrieved by incubation of slides in sub-boiling 10 mM sodium citrate, pH 6.0 for 30 min. To reduce non-specific background sections were incubated with 3 % hydrogen peroxide for 10 min at room temperature. Then each slide containing two or three sections of rat brain was incubated with two different dilutions of primary antibodies and no primary antibodies as a negative control. Primary antibodies against: 8-Hydroxyguanosine (15A3) (dilution 1:400), BiP (GRP78) (dilution 1: 1000), and HSP70 (5A5) (dilution 1:50 for cortex and nucleus accumbens, and 1:100 for brain stem) from Abcam, and actin (dilution 1: 100), ATF4 (dilution 1: 100), P-eIF2α (dilution 1: 100), and PDGFRα (dilution 1: 100) were diluted in SignalStain Antibody diluent (Cell Signaling). Sections were processed with either VectaStain ABC (to detect BiP) or ImmPRESS Anti-Mouse Ig Rat adsorbed (peroxidase) Polymer Detection Kits (to detect 8-Hydroxyguanosine and HSP70) (Vector Laboratories, Inc.). The signal was visualized by DAB-nickel kit (Vector Laboratories, Inc.). The nuclei were stained using the hematoxylin Gill’s 3 formulation (VWR) followed by wash in 0.2 % ammonia water. Of note, that the slides stained with antibodies against 8-Hydroxyguanosine were not counterstained with hematoxylin. The slides were then treated with graded alcohols and xylene. The coverslips were placed using HistoChoice^®^ Mounting Media (VWR). Images were taken using Olimpus BX43F microscope. To analyse the images we used two type of scoring: quick score (Q) and density score. The Q method: immunoreactivity was analyzed visually by calculating the percentage (P) of cells showing estimated intensity (I) of staining (1, weak; 2, moderate; and 3, strong staining). Results are represented as a quick score Q = P × I; maximum 300, as described in http://www.ihcworld.com/ihc_scoring.htm. Density method: immunoreactivity signal was measured by ImageQuant TL software (GE Healthcare Life Science) and then “value” of the signal was divided to the “area” of the image. In both cases results are presented as mean of at least three independent experiments using brains slides from different animal (±SEM). To determine statistical significance, data was analyzed by Student’s t test, and data with p value lower than 0.05 was considered to be statistically different.

### Polysomal ultracentrifugation in sucrose density gradients

The nucleus accumbens lysates from the same drug treatment experiment (W and O1 or O2) were analyzed in each polysomal ultracentrifugation experiment. An equal volume of nucleus accumbens lysate (generally 300 μl) was layered onto a 15–45 % (w/v) sucrose gradient containing 100 μg/ml cycloheximide (VWR) and centrifuged in a Beckman SW41Ti rotor at 38,000 rpm at 4 °C for 2 h. Gradients were collected in 1-ml fractions with continuous monitoring of absorbance at 254 nm using an Isco syringe pump with UV-6 detector (Teledyne Isco Inc.). Samples were stored at −80 °C until further use. The polysomal analysis was repeated three times using lysates from different drug administration experiments.

### Real-time PCR of total and polysomal RNA

Before RNA isolation, 600 μl aliquots from each fraction after polysomal ultracentrifugation in sucrose density gradients were spiked with 100 pg of GFP mRNA (internal control). Then, RNA was purified with TRIzol^®^-LS reagent (Invitrogen) according to the manufacturer’s protocol. The RNA was further precipitated with 0.8 M Na-acetate and 1.2 M NaCl, re-suspended in RNase-free water and precipitated again with 2 M LiCl overnight at −20 °C. Reverse transcription was performed with random primers and reverse transcriptase from the TaqMan^®^ Reverse Transcription Reagents kit (Applied Biosystems) following the manufacturer’s protocol. Quantitative real-time PCR was used to measure the GFP, actin, ATF4 and PDGFRα mRNAs level in each fraction. Amplification and detection were performed using the iCycler IQ Real-time PCR detection system with IQ™ SYBRgreen Supermix (Bio-Rad). The actin, ATF4 and PDGFRα mRNA levels were normalized with the GFP internal control. Relative amount of individual mRNA in each fraction (after normalization to GFP signal) was expressed as a percentage of the sum of this mRNA in all 12 fractions set as 100 %. To assist statistical significance of the changes in the mRNA redistribution along the sucrose density gradients, the percentage of individual mRNA co-sedimented with heavy polyribosomes, containing efficiently translated mRNAs (fractions #7–12), was calculated as a percentage of the total mRNA. The percentage of individual mRNA in polysomal fractions was investigated in three polysomal analyses using lysates from three drug administration experiments. Results are presented as mean values (±SEM). To determine statistical significance, data was analyzed by Student’s t test, and data with p value lower than 0.05 was considered to be statistically different.

### Protein analysis in sucrose gradient fractions

Four hundred μl aliquots from each fraction after polysomal ultracentrifugation in sucrose density gradients was combined with 44 μl 100 % Trichloroacetic acid (TCA) and incubated overnight at 4 °C. After centrifugation at 15,000×*g* for 15 min, pellets were washed twice with cold acetone (−20 °C), dried under vacuum and re-suspended in 100 μl of NuPAGE^®^LDS sample Buffer (Invitrogen). The 20 μl aliquots of fractions 2–11 were loaded on 4–12 % NuPAGE^®^ Novex^®^ Bis–Tris Gel, (Invitrogen) and analyzed as it described in Western Blot Analysis section. The fractions #1 and #12 were excluded from the western blot analyses due to inability to dissolve the pellet after TCA precipitation (fraction #1) and lack of proteins (fraction #12) assessed in a separate experiment.

### MCF7 cell culture, drug treatment and western blot analysis

The MCF7 human breast adenocarcinoma cells (ATCC) were maintained in the Dulbecco’s modified Eagle’s medium (DMEM)/low glucose (Hyclone, Logan, UT) supplemented with 10 % fetal bovine serum and 1 % penicillin–streptomycin (Gibco, Carlsbad, CA). Cells were plated into 100 mm plates at 1.6 × 10^6^ cells per plate and allowed to adhere overnight. Two hours prior to drug treatment, cells were washed with warm PBS and supplemented with fresh media. Prior to oxycodone addition, cells first were pre-incubated with either water or 10 μM of pan opioid-receptor antagonist naloxone hydrochloride (Sigma) for 15 min. After that 5 μM oxycodone or equal volume of vehicle (10 % DMSO in water) were added to the plate. Cells were allowed to grow for the next 24 h and then harvested. To analyze the expression and phosphorylation of proteins, cells were lysed directly on a plate in cold RIPA–EDTA buffer (50 mmol/L Tris–HCl, 150 mmol/L NaCl, 1 % NP-40, 0.5 % sodium deoxycholate, 0.1 % SDS, and 5 mmol/L EDTA, pH 7.4) containing phosphatase inhibitor cocktails 2 and 3 (Sigma), and protease inhibitor (Pierce). Cells were scraped, collected, and then protein concentration was determined using BCA protein assay kit (Pierce, Rockford, IL, USA). Ten micrograms of total protein were resolved on the 4–12 % NuPAGE^®^ Novex^®^ Bis–Tris Gel (Invitrogen) by SDS-PAGE, transferred to a Nitrocellulose membrane (Bio-Rad) and then analyzed by western blotting as it is described for the brain lysate analysis. The hsp70, BiP, and ATF4 signals were normalized to the actin in corresponding sample. The phosphor-eIF2α signal was normalized to the total eIF2α in corresponding sample. To analyze the effect of each treatment on the expression of proteins, normalized signals from oxycodone, naloxone, and naloxone plus oxycodone samples were compared to the corresponding data from the control lysate. To determine statistical significance of the effect caused by the treatment, data from oxycodone or naloxone were compared to the control using Student’s t test, and data with p value lower than 0.05 was considered to be statistically different. To investigate whether oxycodone induces the ISR in the µ-opioid-receptor dependent manner, normalized signals from the cells treated with oxycodone plus naloxone were compared to the data from the oxycodone treated cells using Student’s t test, and data with p value lower than 0.05 was considered to be statistically different. Results are presented as mean values (±SEM) of three independent experiments using different cell cultures. For each experiment, western blot was repeated at least twice.

## Abbreviations

3N: 3-nitrotyrosine
8H: 8-Hydroxyguanosine
ac: anterior commissure
ATF4: cAMP element binding transcription factor 4 or activating transcription factor 4
BBB: blood brain barrier
BiP or GRP78: binding immunoglobulin protein or glucose regulated protein 78
CaMKII: Ca^2+^/calmodulin-dependent protein kinase II
CNS: central nervous system
eIF2α: eukaryotic initiation factor 2 alpha
eIF4A: eukaryotic initiation factor 4A
eIF4E: eukaryotic initiation factor 4E
ER: endoplasmic reticulum
Erk1/2: extracellular signal regulated kinases 1 and 2
Fisher’s PLSD: Fisher’s protected least significant difference
GAPDH: glyceraldehyde 3-phosphate dehydrogenase
GCN2: general control nonderepressible 2 kinase
GL: granular layer
HRI: hemin-regulated inhibitor kinase
hsp70: heat shock protein 70
ISR: integrated stress response
ML: molecular layer
NO: nitric oxide
Nx: naloxone
Oxy: oxycodone
PDGFRα: platelet-derived growth factor receptor alpha
PERK: PKR-like endoplasmic reticulum kinase
PKA: protein kinase A
PKR: protein kinase R
ROS: reactive oxygen species
Ps: polysomes
Rs-L7a: large ribosomal subunit protein 7a
uORF: upstream open reading frame
UPR: unfolded protein response
UTR: untranslated region
WM: white matter

## References

[1] Leppert W (2010). Role of oxycodone and oxycodone/naloxone in cancer pain management. Pharmacol Rep.

[2] Manchikanti L, Helm S, Fellows B, Janata JW, Pampati V, Grider JS, Boswell MV (2012). Opioid epidemic in the United States. Pain Physician.

[3] McDonald DC, Carlson K, Izrael D (2012). Geographic variation in opioid prescribing in the U.S. J Pain.

[4] International Narcotics control board: 2014 report. Estimated world requirements for 2015. Statistics for 2013. Available: https://www.incb.org/incb/en/narcotic-drugs/Technical_Reports/narcotic_drugs_reports.html. Accessed 25 Mar 2015.

[5] Ammon S, Mayer P, Riechert U, Tischmeyer H, Hollt V (2003). Microarray analysis of genes expressed in the frontal cortex of rats chronically treated with morphine and after naloxone precipitated withdrawal. Brain Res Mol Brain Res.

[6] Ammon-Treiber S, Tischmeyer H, Riechert U, Hollt V (2004). Gene expression of transcription factors in the rat brain after morphine withdrawal. Neurochem Res.

[7] Ammon-Treiber S, Hollt V (2005). Morphine-induced changes of gene expression in the brain. Addict Biol.

[8] McClung CA, Nestler EJ, Zachariou V (2005). Regulation of gene expression by chronic morphine and morphine withdrawal in the locus ceruleus and ventral tegmental area. J Neurosci.

[9] Hassan HE, Myers AL, Lee IJ, Chen H, Coop A, Eddington ND (2010). Regulation of gene expression in brain tissues of rats repeatedly treated by the highly abused opioid agonist, oxycodone: microarray profiling and gene mapping analysis. Drug Metab Dispos.

[10] Abul-Husn NS, Annangudi SP, Ma’ayan A, Ramos-Ortolaza DL, Stockton SD, Gomes I, Sweedler JV, Devi LA (2011). Chronic morphine alters the presynaptic protein profile: identification of novel molecular targets using proteomics and network analysis. PLoS One.

[11] Zhang Y, Mayer-Blackwell B, Schlussman SD, Randesi M, Butelman ER, Ho A, Ott J, Kreek MJ (2014). Extended access oxycodone self-administration and neurotransmitter receptor gene expression in the dorsal striatum of adult C57BL/6J mice. Psychopharmacology.

[12] Mayer-Blackwell B, Schlussman SD, Butelman ER, Ho A, Ott J, Kreek MJ, Zhang Y (2014). Self administration of oxycodone by adolescent and adult mice affects striatal neurotransmitter receptor gene expression. Neuroscience.

[13] Przewlocki R (2004). Opioid abuse and brain gene expression. Eur J Pharmacol.

[14] Sanchez-Blazquez P, Rodriguez-Munoz M, Garzon J (2010). Mu-opioid receptors transiently activate the Akt-nNOS pathway to produce sustained potentiation of PKC-mediated NMDAR-CaMKII signaling. PLoS One.

[15] LaLumiere RT, Kalivas PW (2008). Glutamate release in the nucleus accumbens core is necessary for heroin seeking. J Neurosci.

[16] Zhou JF, Yan XF, Ruan ZR, Peng FY, Cai D, Yuan H, Sun L, Ding DY, Xu SS (2000). Heroin abuse and nitric oxide, oxidation, peroxidation, lipoperoxidation. Biomed Environ Sci.

[17] Oliveira MT, Rego AC, Morgadinho MT, Macedo TR, Oliveira CR (2002). Toxic effects of opioid and stimulant drugs on undifferentiated PC12 cells. Ann N Y Acad Sci.

[18] Zhang YT, Zheng QS, Pan J, Zheng RL (2004). Oxidative damage of biomolecules in mouse liver induced by morphine and protected by antioxidants. Basic Clin Pharmacol Toxicol.

[19] Guzman DC, Vazquez IE, Brizuela NO, Alvarez RG, Mejia GB, Garcia EH, Santamaria D, de Apreza MR, Olguin HJ (2006). Assessment of oxidative damage induced by acute doses of morphine sulfate in postnatal and adult rat brain. Neurochem Res.

[20] Xu B, Wang Z, Li G, Li B, Lin H, Zheng R, Zheng Q (2006). Heroin-administered mice involved in oxidative stress and exogenous antioxidant-alleviated withdrawal syndrome. Basic Clin Pharmacol Toxicol.

[21] Ozmen I, Naziroglu M, Alici HA, Sahin F, Cengiz M, Eren I (2007). Spinal morphine administration reduces the fatty acid contents in spinal cord and brain by increasing oxidative stress. Neurochem Res.

[22] Koch T, Seifert A, Wu DF, Rankovic M, Kraus J, Borner C, Brandenburg LO, Schroder H, Hollt V (2009). mu-opioid receptor-stimulated synthesis of reactive oxygen species is mediated via phospholipase D2. J Neurochem.

[23] Skrabalova J, Drastichova Z, Novotny J (2013). Morphine as a potential oxidative stress-causing agent. Mini Rev Org Chem.

[24] Ron D (2002). Translational control in the endoplasmic reticulum stress response. J Clin Invest.

[25] Wek RC, Jiang HY, Anthony TG (2006). Coping with stress: eIF2 kinases and translational control. Biochem Soc Trans.

[26] Hinnebusch AG (2005). Translational regulation of GCN4 and the general amino acid control of yeast. Annu Rev Microbiol.

[27] Vattem KM, Wek RC (2004). Reinitiation involving upstream ORFs regulates ATF4 mRNA translation in mammalian cells. PNAS.

[28] Lu PD, Jousse C, Marciniak SJ, Zhang Y, Novoa I, Scheuner D, Kaufman RJ, Ron D, Harding HP (2004). Cytoprotection by pre-emptive conditional phosphorylation of translation initiation factor 2. EMBO J.

[29] Nestler EJ (1993). Cellular responses to chronic treatment with drugs of abuse. Crit Rev Neurobiol.

[30] Robison A, Nestler E (2011). Transcriptional and epigenetic mechanisms of addiction. Nat Rev Neurosci.

[31] Stetler RA, Gan Y, Zhang W, Liou AK, Gao Y, Cao G, Chen J (2010). Heat shock proteins: cellular and molecular mechanisms in the central nervous system. Prog Neurobiol.

[32] Harding HP, Novoa I, Zhang Y, Zeng H, Wek R, Schapira M, Ron D (2000). Regulated translation initiation controls stress-induced gene expression in mammalian cells. Mol Cell.

[33] Dang Do AN, Kimball SR, Cavener DR, Jefferson LS (2009). eIF2{alpha} kinases GCN2 and PERK modulate transcription and translation of distinct sets of mRNAs in mouse liver. Physiol Genom.

[34] Batra VR, Schrott LM (2011). Acute oxycodone induces the pro-emetic pica response in rats. J Pharmacol Exp Ther.

[35] Davis CP, Franklin LM, Johnson GS, Schrott LM (2010). Prenatal oxycodone exposure impairs spatial learning and/or memory in rats. Behav Brain Res.

[36] Rodriguez Parkitn JM, Bilecki W, Mierzejewski P, Stefanski R, Ligeza A, Bargiela A, Ziolkowska B, Kostowski W, Przewlocki R (2004). Effects of morphine on gene expression in the rat amygdala. J Neurochem.

[37] Ammon-Treiber S, Grecksch G, Stumm R, Riechert U, Tischmeyer H, Reichenauer A, Hollt V (2004). Rapid, transient, and dose-dependent expression of hsp70 messenger RNA in the rat brain after morphine treatment. Cell Stress Chaperones.

[38] Cui J, Wang Y, Dong Q, Wu S, Xiao X, Hu J, Chai Z, Zhang Y (2011). Morphine protects against intracellular amyloid toxicity by inducing estradiol release and upregulation of Hsp70. J Neurosci.

[39] Chu NN, Xia W, Yu P, Hu L, Zhang R, Cui CL (2008). Chronic morphine-induced neuronal morphological changes in the ventral tegmental area in rats are reversed by electroacupuncture treatment. Addict Biol.

[40] Su EJ, Fredriksson L, Geyer M, Folestad E, Cale J, Andrae J, Gao Y, Pietras K, Mann K, Yepes M (2008). Activation of PDGF-CC by tissue plasminogen activator impairs blood-brain barrier integrity during ischemic stroke. Nat Med.

[41] Ma Q, Huang B, Khatibi N, Rolland W, Suzuki H, Zhang JH, Tang J (2011). PDGFR-alpha inhibition preserves blood-brain barrier after intracerebral hemorrhage. Ann Neurol.

[42] Wen H, Lu Y, Yao H, Buch S (2011). Morphine induces expression of platelet-derived growth factor in human brain microvascular endothelial cells: implication for vascular permeability. PLoS One.

[43] Sharma HS, Ali SF (2006). Alterations in blood-brain barrier function by morphine and methamphetamine. Ann N Y Acad Sci.

[44] Sharma HS, Sjoquist PO, Ali SF (2010). Alterations in blood-brain barrier function and brain pathology by morphine in the rat. Neuroprotective effects of antioxidant H-290/51. Acta Neurochir Suppl.

[45] Beauvais G, Atwell K, Jayanthi S, Ladenheim B, Cadet JL (2011). Involvement of dopamine receptors in binge methamphetamine-induced activation of endoplasmic reticulum and mitochondrial stress pathways. PLoS One.

[46] Green TA, Alibhai IN, Unterberg S, Neve RL, Ghose S, Tamminga CA, Nestler EJ (2008). Induction of activating transcription factors (ATFs) ATF2, ATF3, and ATF4 in the nucleus accumbens and their regulation of emotional behavior. J Neurosci.

[47] Liu J, Pasini S, Shelanski ML, Greene LA (2014). Activating transcription factor 4 (ATF4) modulates post-synaptic development and dendritic spine morphology. Front Cell Neurosci.

[48] Kiyatkin EA, Sharma HS (2009). Acute methamphetamine intoxication: brain hyperthermia, blood-brain barrier, brain edema, and morphological cell abnormalities. Int Rev Neurobiol.

[49] Kousik SM, Napier TC, Carvey PM (2012). The effects of psychostimulant drugs on blood brain barrier function and neuroinflammation. Front Pharmacol.

[50] Costa-Mattioli M, Sossin WS, Klann E, Sonenberg N (2009). Translational control of long-lasting synaptic plasticity and memory. Neuron.

